# Three factor eating questionnaire-R18 as a measure of cognitive restraint, uncontrolled eating and emotional eating in a sample of young Finnish females

**DOI:** 10.1186/1479-5868-6-41

**Published:** 2009-07-17

**Authors:** Susanna Anglé, Janne Engblom, Tiina Eriksson, Susanna Kautiainen, Marja-Terttu Saha, Pirjo Lindfors, Matti Lehtinen, Arja Rimpelä

**Affiliations:** 1 School of Public Health, University of Tampere, Tampere, Finland; 2 Turku School of Economics, Turku, Finland; 3 Tampere University Hospital, Department of Paediatrics, University of Tampere, Tampere, Finland

## Abstract

**Background:**

The aim of the study was to examine the construct validity of the Three-Factor Eating Questionnaire -R18 (TFEQ-R18), a measure of eating behaviour, and to evaluate cognitive restraint, uncontrolled eating and emotional eating in a sample of adolescent and young adult females of different weights.

**Methods:**

Subjects were 2 997 females, aged 17 to 20 years, who participated in a phase III human papillomavirus vaccination trial in Finland in 2004 – 2009.

Self-administered questionnaires and weight and height measurements were used. The factor structure of the TFEQ-R18 was verified by factor analysis. Connections between measured eating behaviour and Body Mass Index (BMI) were tested using analysis of variance.

**Results:**

The original factor structure of the TFEQ-R18 was replicated: six of the eighteen items measured cognitive restraint, nine measured uncontrolled eating, and three measured emotional eating. On average, higher BMI was associated with higher levels of cognitive restraint (p < 0.001) and emotional eating (p < 0.001), but not with uncontrolled eating.

**Conclusion:**

Structural validity of the TFEQ-R18 was good in this sample of young Finnish females with a varying range of body weights. Use of the instrument as a measure of eating behaviour was thus corroborated. Connections of restrained and emotional eating with BMI were in accordance with previous findings from young females.

## Background

The psychology of eating behaviour – e.g. the cognitive, behavioural and emotional aspects of eating habits – call for more attention given the increasing prevalence of obesity and other eating related problems. The prevalence of overweight and obesity has increased world wide [1]. Disordered eating in different forms and of various degrees of severity are common, especially among adolescent girls and young women [2]. There is a need for valid and usable instruments for evaluating eating behaviour, applicable to a variety of populations.

One of the most widely used measures in the field of eating behaviour research is the Three Factor Eating Questionnaire, TFEQ, developed by Stunkard and Messick [3]. Originally, this self-assessment questionnaire was designed to measure cognitive and behavioural components of eating in obese populations. It contains 51 items, aggregated into three scales: "Cognitive Restraint", "Disinhibition" and "Hunger".

However, some findings have raised concern about the factor structure and the factor stability of the 51-item TFEQ [4], [5], [6]. Karlsson and colleagues [6] evaluated scaling properties and construct validity of the TFEQ in a sample of 4 377 Swedish, middle-aged, obese men and women, and found that the original factor structure of TFEQ was not replicated. Based on psychometric analyses, a revised version of the questionnaire was constructed, consisting of three factors: "Cognitive Restraint", "Uncontrolled Eating" and "Emotional Eating". Using the most efficient items to boost the convergent and discriminant validity of the new scales eventually led to a revised, shorter, 18-item version of the instrument, the Three Factor Eating Questionnaire – R18 (TFEQ-R18) [6].

The TFEQ-R18 was a step forward in the psychometrics of eating behaviour. Although it was constructed using data from obese adults, it has been shown to be applicable to other populations as well [7,8]. Among French adolescents and adults, the TFEQ-R18 was found to be easy and comprehensible to the respondents, and able to distinguish among different eating patterns in a general population [8].

The concepts of cognitive restraint, uncontrolled eating and emotional eating, corresponding to the three factors of the TFEQ-R18, originate from obesity research, but none of the behaviours are characteristic of obese populations only. The term restraint ("dietary restraint"; "restrained eating"; or "cognitive restraint") has been one of the most central and debated concepts in the study of human eating behaviour since the restraint theory of obesity [9,10]. Restraint refers to a tendency to constantly and consciously restrict one's food intake instead of using physiological cues, hunger and satiety, as regulators of food intake. Restrained eating, however, is not the same thing as dieting [11], [12], [13]. Rather, restrained eaters consume less food than they would like to eat – but not necessarily less than they need to maintain energy balance [14]. Uncontrolled eating refers to a tendency to overeat, with the feeling of being out of control. Emotional eating means the tendency to eat in response to negative emotions.

The aim of the present study was to evaluate whether the factor structure of the TFEQ-R18 as described by Karlsson et al. [6] would be replicated in a Finnish sample of adolescent and young adult females representing different weight categories. We also examined connections between body weight and TFEQ-R18 -scores in this population.

## Methods

### Subjects

Data from 2 997 girls and young women, aged 17 to 20 years (mean 18.6 y), were analysed. Subjects were recruited among the Finnish participants in a phase III human papillomavirus (HPV-16/18) vaccination trial (HPV-008). The trial aims at examining efficacy of a vaccine against human papillomavirus [15], and will follow the long-term efficacy of the vaccine against cervical carcinoma [16]. Originally, as described in detail by Lehtinen et al. [16], 24 046 girls, aged 16–17 years, from 18 cities in Finland were invited to the phase III HPV vaccination trial, between May 2004 and June 2005. A total of 4 808 subjects volunteered. Fifteen of the 18 vaccination centres in the multi-center trial operated under Tampere University (in the cities of Espoo, Jyväskylä, Järvenpää, Kotka, Kouvola, Kuopio, Lahti, Lappeenranta, Mikkeli, Pori, Rauma, Seinäjoki, Tampere, Turku, and Vaasa). Participants of the present study were recruited among the 3 515 subjects in these 15 centres.

Between September 2006 and December 2007, during their routine six-monthly follow-up visits to the 15 vaccination centres, the participants were offered the possibility to take part in a vaccination centre – randomized study promoting their health habits. A total of 3 070 subjects (87.3% of the 3 515 invitees) gave their informed consent and received self-report questionnaires on eating behaviour and health habits. Finally, 3 002 subjects (85.4%) returned the questionnaire. After exclusion of five subjects due to pregnancy, the final sample consisted of 2 997 girls and young women (85.3% of all invitees). Most (61.8%) were full-time students. According to the reports of the subjects, 28.2% had fathers with 12 or more years of schooling, and 44.2% had mothers with 12 or more years of schooling.

Complete weight and height data were available from 2 943 subjects. Mean weight was 63.1 (SD 0.2) kg, ranging from 38.4 to 131.3 kg. Mean height was 165.8 (SD 0.1) cm, and height ranged from 137.5 to 199.5 cm. Body Mass Index (BMI) was on average 22.9 (SD 0.1) kg/m^2^. BMI ranged between 14.4 and 49.8 kg/m^2^. The proportion of underweight (BMI < 18.5) was 6.3% (n = 184). The majority, 72.0% (n = 2 120) were of normal weight (BMI 18.5 – 24.9). BMI of 16.4% of subjects (n = 482) were in the overweight range (BMI 25.0 – 29.9). Five-point-three percent (n = 157) of subjects filled the criteria of obesity (BMI ≥ 30.0).

### Measures

#### Three Factor Eating Questionnaire – R18

The Finnish version of the TFEQ-R18 has been translated and back-translated from English by the Finnish Association for the Study of Obesity. For the present study, we made one small change to one of the questionnaire items. In Finland, among adolescent and young adult females, steaks and meat are not considered the most desired or tempting foods. Rather, e.g. vegetarianism has become more and more common [17]. Thus, we modified item 1, *"When I smell ****a sizzling steak or a juicy piece of meat***, *I find it very difficult to keep from eating, even if I have just finished a meal."*, into *"When I smell ****a delicious food***, *I find it very difficult to keep from eating, even if I have just finished a meal." *It was considered that this slight change would improve the ability of the statement to measure – in this population – what it was originally meant to measure: difficulty controlling eating when tempted by external stimuli, even when already full. To keep the change as neutral as possible, the original, vivid description was replaced simply with a more general expression. As recommended by the designers of the TFEQ-R18, responses to all 18 items were coded on a four-point scale (1 – 4), with higher values indicating more of the behaviour. The four-point response alternatives measured, e.g., how frequent was a certain behaviour, or how true a statement was for the respondent [6]. Brief questions on schooling of the respondent and her parents were included in the questionnaire.

#### Anthropometric measurements

Body weight of each subject was measured in underwear to the nearest 0.1 kg using electronic scales. In every study centre, identical, calibrated scales were used. Standing height was measured to the nearest 0.5 cm. Body Mass Index (BMI), weight in kg divided by height in m squared, was calculated.

### Study protocol

Subjects in phase III of the HPV vaccination trial visited the vaccination centers of Tampere University biannually. Beginning in September 2006, they received invitation letters to our study on eating behaviour and health habits, and questionnaires. Letters were mailed about two weeks prior to a scheduled, six-monthly HPV study visit of each subject. During the visit the subjects returned the filled questionnaires, and their weights and heights were measured by study nurses.

### Ethical considerations

Both the phase III HPV vaccination trial, and use of the questionnaire of the present health promotion study, were approved by the National Advisory Board on Health Care Ethics (ETENE), Sub-Committee on Medical Research Ethics (TUKIJA). Separate informed consent (for both the vaccination trial and the health promotion study) was obtained from all subjects.

### Statistical Analyses

The SPSS programme, version 15.0, was used in performing the statistical analyses. The factor structure of the TFEQ-R18 -questionnaire was explored using Principal Components Analysis with a Varimax rotation. A cut-off point of <0.30 was used for the factor loadings. Before examining the connections between TFEQ-R18 scores and BMI, raw scores of Cognitive Restraint, Uncontrolled Eating and Emotional Eating were converted, as recommended in the scoring instructions of TFEQ-R18. The converted scores represent the relative proportion (%) of highest possible raw scores, ranging from 0 to 100:



In the formula, S stands for raw score, L stands for lowest possible raw score, and R_s _stands for possible raw score range.

Connections between the converted Cognitive Restraint, Uncontrolled Eating and Emotional Eating scores and classified BMI were examined using one-way ANOVAs. In one-way ANOVAs, populations are assumed to be normally distributed and homoscedastic with independent observations. In this study the normality assumption was not required because of large sample sizes (based on the central limit theorem). The assumption of homoscedasticity was examined with Levene's test of equal variances and ANOVAs were carried out accordingly. Correlations between study variables were analysed using Pearson Correlation Coefficients.

## Results

### Factor structure of the TFEQ-R18

According to the principal component analysis with a Varimax rotation, the original factor structure of the TFEQ-R18 was replicated in our sample. The factor analysis produced item communalities ranging from 0.34 to 0.84 (Table 1). Communalities of 15 of the 18 items were above 0.40. The analysis proposed a three component model, which explained 54.2% of the total variance. A rotated component matrix revealed that each of the 18 items strongly loaded positively to one of the three factors. Using different factor analyses and using different rotations (Promax, Oblimin) produced similar factor structures and high item loadings, thus corroborating the results.

**Table 1:** Factor loadings of the TFEQ-R18 items. Loadings < 0.30 are left blank.

**Item**		**Uncontrolled** **Eating**	**Cognitive** **Restraint**	**Emotional** **Eating**	**Communality**
8	I get so hungry that my stomach often seems like a bottomless pit.	0.76			0.47
9	I am always hungry so it is hard for me to stop eating before I finish the food on my plate.	0.73			0.60
1	When I smell a delicious food, I find it very difficult to keep from eating, even if I have just finished a meal.*	0.68			0.78
17	Do you go on eating binges though you are not hungry?	0.68			0.54
4	Sometimes when I start eating, I just can't seem to stop.	0.65			0.34
13	I am always hungry enough to eat at any time.	0.65			0.84
7	When I see a real delicacy, I often get so hungry that I have to eat right away.	0.62			0.43
14	How often do you feel hungry?	0.59			0.60
5	Being with someone who is eating often makes me hungry enough to eat also.	0.57			0.57
11	I consciously hold back at meals in order not to gain weight.		0.81		0.74
2	I deliberately take small helpings as means of controlling my weight.		0.77		0.66
12	I do not eat some foods because they make me fat.		0.73		0.54
18	On a scale of 1 to 8, where 1 means no restraint in eating and 8 means total restraint, what number would you give yourself?		0.71		0.43
16	How likely are you to consciously eat less than you want?		0.65		0.38
15	How frequently do you avoid 'stocking up' on tempting foods?		0.58		0.36
6	When I feel blue, I often overeat.			0.87	0.42
3	When I feel anxious, I find myself eating.			0.85	0.51
10	When I feel lonely, I console myself by eating.			0.81	0.55

*Item 1 has been slightly modified to better fit the target population of the present study. The original statement was about smelling "a sizzling steak or a juicy piece of meat"; but these are not typically considered delicate or tempting among young, Finnish females.

The factor structure obtained corresponded the structure of the original TFEQ-R18. Thus, six of the 18 items measured cognitive restraint, nine items measured uncontrolled eating, and three items measured emotional eating, among adolescent and young adult, Finnish females. Cronbach's alphas of these three scales were high: 0.75 for cognitive restraint, 0.85 for uncontrolled eating, and 0.87 for emotional eating.

### Connections between TFEQ-R18 scores and BMI

When examining BMI as a continuous variable, BMI and TFEQ-R18 -scores were correlated among adolescent females: the higher the BMI, the higher the cognitive restraint score (r = 0.28, p < 0.001) and the higher the emotional eating score (r = 0.20, p < 0.001). The positive correlation between BMI and uncontrolled eating was weak, but statistically significant (r = 0.063, p < 0.001).

The results were quite similar when BMI was analysed by category, dividing the subjects into the underweight (BMI <18.5), the normal weight (BMI 18.5 – 24.9), the overweight (BMI 25.0 – 29.9), and the obese (BMI ≥ 30.0). Between these four BMI categories, the mean scores of cognitive restraint and emotional eating differed significantly (Table 2). The connection between emotional eating and classified BMI was straight-forward: mean score of emotional eating was lowest in the underweight category, and highest in the overweight category, and pair-wise differences between the four BMI categories were all statistically significant. Also the level of cognitive restraint increased with increasing BMI. Pair-wise comparisons of restraint scores between the BMI categories reached statistical significance, except for the comparison between overweight and obesity. The mean scores of uncontrolled eating did not differ significantly between the BMI categories.

**Table 2:** TFEQ-R18 -scores (mean, sd) in the weight categories of underweight (I), normal weight (II), overweight (III) and obese (IV).

	**I** BMI < 18.5 *n *= 181	**II** BMI 18.5–24.9 *n *= 2 074	**III** BMI 25.0–29.9 *n *= 469	**IV** BMI ≥ 30.0 *n *= 154	**p-value†**	**Pairwise comparisons**
**Cognitive** **Restraint**	19.5 (18.2)	33.7 (18.7)	41.2 (15.7)	42.6 (15.2)	<0.001	I vs II** I vs III** I vs IV** II vs III** II vs IV** III vs IVns
						
**Uncontrolled** **Eating**	30.7 (16.9)	32.3 (17.0)	33.3 (17.3)	35.4 (15.8)	0.05	All pairwise comparisons NS
						
**Emotional** **Eating**	22.5 (22.2)	30.3 (25.7)	37.6 (28.8)	46.6 (28.7)	<0.001	I vs II** I vs III** I vs IV** II vs III** II vs IV** III vs IV*

Cognitive Restraint, Uncontrolled Eating and Emotional Eating each get scores from 0 to 100, with higher values indicating more of the behaviour. (N = 2 878)

† = One way ANOVA, * = p < .05, ** = p < .001

## Discussion

In the present study, factor structure and construct validity of a brief measure of eating behaviour, the Three Factor Eating Questionnaire Revised -18 (TFEQ-R18) [6], was examined in a population-based sample of Finnish adolescent and young adult females [16]. Originally, based on psychometric analyses using data from Swedish obese men and women, Karlsson and colleagues shortened and revised the 51-item-Three Factor Eating Questionnaire [3], into a version with 18 items [6]. Our analyses of TFEQ-R18 -data from the 2 997 young Finnish females with varying body weights produced a factor structure that corresponded to the one found by Karlsson et al [6]: six items loaded high on the factor "Cognitive Restraint", nine items loaded high on "Uncontrolled Eating", and three items loaded high on "Emotional eating". Construct validity of the TFEQ-R18 was good.

Our findings corroborate earlier results suggesting that the TFEQ-R18 is a valid measure of eating behaviour not only in the obese but also in the general population. In samples of French adolescents and adults, multitrait/multi-item scaling analyses showed satisfactory internal consistency of the French translation of TFEQ-R18 [8]. The French study reported very similar internal consistency reliability coefficients (Cronbach's alphas) for the three scales to the ones reported by the designers of the TFEQ-R18 [6].

### TFEQ-R18 scores and body weight

The secondary aim of our study was to analyse connections between body weight and TFEQ-R18 scores. Connections between eating behaviour and body weight have been extensively studied, especially in the field of obesity research. However, to the best of our knowledge, there are but two earlier studies that explored this connection in samples with varying weights, and in which TFEQ-R18 was used as a measure of eating behaviour. Elfhag and Linné studied eating behaviour and relative weight of Swedish women, aged 35 to 65 years, and their adolescent children, aged 15 to 18 years [7]. De Lauzon-Guillain and colleagues analysed TFEQ-R18 responses and several measures of adiposity in a sample of French adults and adolescents, over a two-year period [18]. In the latter report, the main focus was on cognitive restraint.

We found that of the three factors of the TFEQ-R18 questionnaire, cognitive restraint and emotional eating were connected with body weight in girls and young women. Higher scores of cognitive restraint and higher scores of emotional eating were associated with a higher BMI. These results are similar to the cross-sectional findings of Elfhag and Linné [7]: both cognitive restraint and emotional eating were positively correlated with BMI in adolescent girls, in adolescent boys, and in their mothers.

Of the three factors of TFEQ-R18, we found no connection between uncontrolled eating and body weight, when BMI was analysed as a categorised variable. When analysing BMI as a continuous variable, there was a statistically significant (p < 0.001), but a very weak (r = 0.063) positive correlation between uncontrolled eating score and BMI. In the latter analysis, the correlation probably reached significance due to the large sample size. In the Swedish adolescents, uncontrolled eating had no connection with BMI, whereas in the adult women, there was a positive correlation between BMI and uncontrolled eating [7]. Our sample was on average slightly older than the girls, but clearly younger than the women of the Swedish study. Perhaps the connection between higher body weight and higher level of uncontrolled eating in females starts to appear with increasing age.

These cross-sectional findings raise the question of whether it is the eating behaviour that predicts body weight, or vice versa. Do the tendencies of cognitive restraint and emotional eating make people gain weight. Or does a higher body weight make people eat differently? The two-year-follow-up by de Lauzon-Guillain and colleagues [18] suggest that the latter might be true, at least in case of cognitive restraint. In the French sample, initial scores of cognitive restraint were not connected with subsequent adiposity changes, neither in adults, nor in adolescents. However, in all age groups studied, higher values of initial adiposity – BMI, waist circumference, sum of skinfold thicknesses, and percentage body fat – predicted a larger increase in cognitive restraint score over the two-year period [18]. Thus, differences in adiposity level seem to *precede *differences in cognitive restraint.

Since the present study was carried out among participants of a phase III human papillomavirus (HPV) vaccination trial, which aims at following the efficacy of a vaccine against the HPV virus and the later development of cervical carcinoma, representativeness of our sample is limited even if the invitation was population-based. The subjects had volunteered to participate in the vaccination trial as well as in our study on eating behaviour. Thus, these young females are a selected group, likely to be more health conscious than their peers among the general population. On the other hand, an ongoing questionnaire study with 4 438 respondents found that the young women who participated in the HPV vaccination trial did not differ significantly from young women who did not participate, regarding certain measures of living conditions and quality of life (e.g. physical functioning and well being, emotional wellbeing, social functioning, life habits and sexual health) (Woodhall et al., unpublished data). Therefore, all in all, the phase III HPV vaccination trial provided a unique opportunity for us to attain a large and representative sample of young females from different parts of Finland. For example, well-organised weight and height measurements of the nearly three thousand subjects by professional nurses – instead of having to settle with self-reported weight data – would hardly have been possible without the organisation of the HPV vaccination trial.

We also slightly modified one of the items of the original TFEQ-R18 questionnaire. The question *"When I smell ****a sizzling steak or a juicy piece of meat***, *I find it very difficult to keep from eating, even if I have just finished a meal." *(item 1) was replaced with *"When I smell ****a delicious food***, *I find it very difficult to keep from eating, even if I have just finished a meal." *The item is supposed to measure the tendency to uncontrolled eating in the absence of hunger, when tempted by external stimuli. In the Finnish culture, steaks and meat are not necessarily considered the most desired foods – definitely not among girls and young women. Steaks are thus a poor example of a tempting food in this population, and the original item needed to be changed in order for it to produce valid responses. The data we gathered using the TFEQ-R18 behaved in the analyses in a very similar manner when compared to earlier analyses of TFEQ-R18 data [6,8,18], suggesting that the instrument was valid despite the slight modification of one of the eighteen items.

## Conclusion

As the construct validity of the TFEQ-R18 appeared to be good in this sample of young Finnish females with a varying range of body weights, usability of the instrument as a measure of eating behaviour was corroborated. Connections of the three factors of the questionnaire with body weight were in accordance with previous findings: higher levels of cognitive restraint and emotional eating were associated with higher BMI. Our results suggest that the TFEQ-R18 is a psychometrically sound and valid measure of the tendencies of cognitive restraint, uncontrolled eating and emotional eating also in Finland, at least among adolescent and young adult females.

## Competing interests

The authors declare that they have no competing interests.

## Authors' contributions

All authors approved the submission of this paper. SA wrote the first draft of the manuscript and it was revised by all eight authors. SA, SK, MTS, PL and AR conceptualized and designed the study. JE designed and conducted the statistical analyses. TE, as the coordinator of the human papillomavirus vaccination trial (HPV 008), organized the recruitment of study participants and data collection of this study. ML, the principal investigator of the HPV 008 trial, contributed to the design of the study, and critical drafting of the manuscript. All authors have read and approved the final manuscript.
